# Potential therapeutic effects of shrimp protein hydrolysates on NAFLD-induced infertility disorders: Insights into redox balance, heat shock protein expression, and chromatin compaction in male rats

**DOI:** 10.22038/ijbms.2024.76649.16589

**Published:** 2025

**Authors:** Somayyeh Rahmani, Ebrahim Najdegerami, Mazdak Razi, Mehdi Nikoo

**Affiliations:** 1 Department of Biology, Faculty of Science, Urmia University, Urmia, Iran; 2 Division of Comparative Histology and Embryology, Department of Basic Science, Faculty of Veterinary Medicine, Urmia University, Urmia, Iran; 3 Artemia & Aquaculture Research Institute, Urmia University, Urmia, Iran

**Keywords:** Heat shock protein, Infertility, NAFLD, Oxidative stress, Protein hydrolysates, Transitional protein

## Abstract

**Objective(s):**

Nonalcoholic fatty liver disease (NAFLD) is known to disrupt testicular anti-oxidant capacity, leading to oxidative stress (OS) that can negatively affect male fertility by damaging sperm DNA. Heat shock proteins (HSP70 and HSP90), in association with transitional proteins (TP1 and TP2), play crucial roles in protecting sperm DNA integrity in oxidative conditions. Whiteleg shrimp protein hydrolysates (HPs) exhibit anti-oxidant properties, prompting this study to explore the potential of HPs in ameliorating NAFLD-induced testicular damage.

**Materials and Methods:**

The study divided rats into four groups: control, a group subjected to a high-fat diet (HFD) to induce NAFLD without supplementation, and two HFD-induced NAFLD groups receiving HP doses (20 and 300 mg/kg). After 70 days, the testicular total anti-oxidant capacity (TAC), malondialdehyde (MDA), glutathione (GSH), glutathione disulfide (GSSG), HSP70-2a, HSP90 expression, and TP mRNA levels were assessed.

**Results:**

The results showed that HFD-induced NAFLD significantly increased GSH and MDA levels and disrupted the GSH/GSSG ratio (*P*<0.05) while also reducing HSP70-2a, HSP90, TP1, and TP2 expression (*P*<0.05). However, HP administration effectively restored testicular redox balance, reduced oxidative stress, and enhanced these protective proteins’ expression compared to HFD (*P*<0.05).

**Conclusion:**

NAFLD negatively affects the testicular redox system and HSP and TP expression, disrupting male fertility potential. In contrast, HP-treated rats showed a marked effect on NAFLD-induced damage by improving testicular anti-oxidant status and regulating the expression of HSPs and TP proteins. These findings suggest a potential therapeutic role for HP in safeguarding male fertility against the damaging effects of NAFLD.

## Introduction

Male-related infertility disorders have been shown to positively correlate with disrupted oxidant/anti-oxidant balance (1). Accordingly, the consequent disruption of enzymatic and nonenzymatic anti-oxidants in testicles can lead to a significant accumulation of reactive oxygen species (ROS) and vulnerable oxidative stress (OS). Among different molecular and ionic anti-oxidant elements, a maintained redox balance is crucial for biological processes related to male gonads (2). Accordingly, redox enzymes, including superoxide dismutase (SOD), glutathione peroxidase (GPX), and glutathione reductase (GR), are actively involved in neutralizing superoxide and hydrogen peroxides (the main ROS in the testicles) through the maintenance of the reduced glutathione (GSH) to oxidized glutathione (GSSG) conversion system (3). Indeed, in addition to keeping cells in a reduced state, glutathione also acts as an electron donor for other anti-oxidative enzymes, including glutathione-S-transferase (GST), as well as a catalyst for conjugating harmful endogenous and exogenous compounds (3, 4). Thus, the disruption of the GSH/GSSG balance can result in the massive production of endogenous peroxidases (delivered from aerobic respiration), which in turn can negatively affect cellular antioxidant levels (5, 6).

Nonalcoholic fatty liver disease (NAFLD) is known to be associated with OS, chronic inflammation, and apoptosis (2). The interplay between OS and inflammation creates a vicious cycle that leads to the apoptosis of testicular germ cells (2). Indeed, not only specific to testicular tissue, increased OS (2), chronic inflammation (7), and apoptotic reactions (8) in various organs are correlated with NAFL. In line with this issue, it is well established that fat redistribution and systemic inflammation in the liver, further affecting sperm quality (9), can disrupt the Sertoli cell’s physiological interactions in maintaining metabolomics (glucose and lactate) production/transition in the testicles (10). These changes can negatively affect the oxidant/anti-oxidant balance via different mechanisms. For instance, chronic inflammatory reactions through overexpression of cytokines and triggering poly-morpho-nuclear immune cells over infiltration (11, 12) and metabolic disorders by altering pentose phosphate flux balance (13) can disrupt the GSH/GSSG balance, initiating OS development in the testicles (14). Numerous studies have demonstrated that the continuous generation of reactive oxygen species (ROS), coupled with an imbalance between oxidants and anti-oxidants, can have a significant impact on the integrity of DNA in both germ cells and sperm cells (15-17)

During the postmeiotic maturation phase, the composition and compactness of chromatin in haploid spermatids undergo a series of dynamic changes. This process involves the transformation of protamine-based chromatin arrays (18). It initiates modifications in the composition of the histone, facilitated by transition proteins 1 and 2 (TP-1, TP-2). These modifications ultimately result in the replacement of histones with protamine proteins, completing the transition that significantly increases chromatin compaction. Therefore, increased compaction serves as a protective mechanism, effectively safeguarding the genome against oxidative damage (19).

In line with this issue, all cell types express the broad family of structurally diverse proteins known as heat shock proteins (HSPs). Indeed, based on their molecular weight and structural properties, HSPs are divided into 20 families (20). In addition to other physiological roles in various cellular signaling pathways, HSPs actively interact in nuclear chromatin condensation-related epigenetic events (21, 22). In testicular tissue, HSP70-2a and, to a lesser extent, HSP90 plays a significant role in regulating and assisting the intricate chromatin remodeling process during genomic maturation in haploid germinal cells (19, 23). Accordingly, HSP70-2a is impressively expressed in the postmeiotic stages to interact in the transition process (24), and HSP90, another member of this family, is highly expressed in myoid, Leydig, and Sertoli cells to maintain mostly the physiological functions of somatic cells (25). Furthermore, HSP90 works in collaboration with HSP70-2a to aid in protein folding and compensates for the diminished function of HSP70-2a during the later stages of spermatogenesis. This cooperative action between HSP90 and HSP70-2a ensures proper protein folding and maintenance of cellular integrity during the critical phases of spermatid development (26). Apart from its mentioned functions, HSP90 also plays a crucial role in assisting other proteins involved in transporting histones from the cytoplasm to the nucleus during the development of spermatocytes. This contribution ensures the proper localization and incorporation of histones into chromatin, thereby facilitating the establishment of proper chromatin structure and gene regulation during spermatocyte development (26, 27). Furthermore, research has demonstrated that HSP70 plays a crucial role in regulating the cellular redox status through its influence on the activities of glutathione-related enzymes. This particular mechanism is believed to be significant in the cytoprotective effects exerted by HSP70 (28).

Studies have demonstrated that certain natural substances, such as bioactive peptides (protein hydrolysate), possess DNA protective effects and anti-oxidant and fat-reducing properties (29, 30). These peptides typically consist of 2 to 20 amino acids and are absorbed by the intestines and circulated in the bloodstream to exert their physiological effects on target tissues. They contribute to human well-being through various mechanisms, including inflammation reduction, blood pressure regulation, anti-obesity effects, and preventing and improving diabetes-related symptoms (31). These proteins play a crucial role in scavenging free radicals and terminating the chain reactions associated with OS (32). Notably, marine-derived hydrolysate proteins with anti-oxidant properties have shown positive effects in various fields of biological sciences. The present research utilized whiteleg shrimp (*Litopenaeus vannamei*), a commonly cultivated species worldwide. Processing waste generated from this species and other shrimp species intended for export accounts for a substantial proportion (50%) of the total product. These waste materials are rich in protein and other valuable substances (pigments, chitin, etc.). In the past, these wastes were often discarded into the environment as they served no purpose and lacked efficiency, leading to physiological and health-related pollution. However, with advancements in food processing technology and the discovery of valuable bioactive compounds, these waste products are now being utilized across various industries. Considering the abundance of aquatic resources in seas and water bodies and the anti-oxidant and redox system-modulating properties exhibited by these waste materials, their appropriate utilization can be regarded as a potential treatment method to mitigate reactive ROS formation in various disorders. Therefore, based on the potential anti-oxidant properties of protein hydrolysate derived from waste materials, as well as the observed connection between chromatin condensation and oxidative damage following disturbances in HSPs (70-2a and 90) and TPs (1 and 2) (19, 23), our current experimental study aimed to investigate the protective effects of protein hydrolysate from Whiteleg shrimp waste. Specifically, we focused on its impact on the histone/protamine transition process by examining its influence on the expression levels of HSP70-2a and HSP90. Additionally, we analyzed the correlation between these expression levels and the balance of GSH/GSSG in rats induced with NAFL.

## Materials and Methods

***Protein hydrolysate preparation***

**Figure 1:**
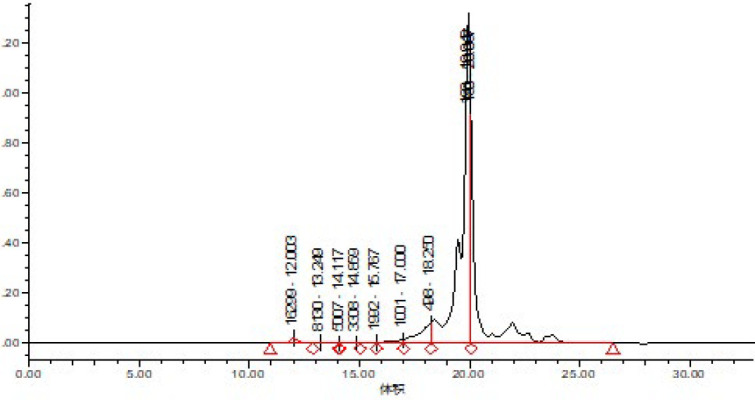
Molecular weight distribution (MWD) of white shrimp protein hydrolysates obtained through alcalase hydrolysis

To obtain protein hydrolysate (HP) from Whiteleg shrimp waste, a mixture of the waste paste and distilled water was prepared in a 1:1 ratio. The mixture was homogenized using a homogenizer (Heidolph DIAX900, Heidolph Instruments GmbH, Schwabach, Germany) for 2 min. Hydrolysis was conducted following the method described by Nikoo *et al*. (2021), with an initial pH of 7.1 and a temperature of 50 °C maintained for 3 hr. Throughout the enzymatic reaction, continuous stirring was applied using a mechanical stirrer (FTDS-11, Sci Finetech Co., Seoul, South Korea). The solution was heated for 10 min at 95 °C to halt the enzymatic reaction. Subsequently, primary filtering was performed after the solution cooled to room temperature using a net cloth. Furthermore, centrifugation was carried out at 4000 rpm for 15 min at 4 °C using a centrifuge (Hermle Labortechnik GmbH, Wehingen, Germany). The resulting liquid was then subjected to freeze drying for 24 hr using a freeze dryer (Christ, Osterode, Germany). When the molecular weight distribution of the resulting protein hydrolysates was examined, it became clear that autolysis had formed peptides with various chain lengths. The data analysis revealed that 40% of the total peptides in the protein hydrolysate were peptides with a molecular weight of less than 500 Da (33) (Figure 1).

***Animals and experimental design***

The animal house of the biology department at Urmia University (Iran) provided 24 male Wistar rats, each weighing an initial average of 230.2 ± 23 g. For each treatment group, there were four cages with six rats each. The rats were fed a regular meal during the adaptation period and housed at a temperature of 25 °C with a 12-hour light cycle. The rats were split into four experimental groups after one week of acclimatization: Control (standard diet), HFD-sole (high-fat diet), HFD+HP(20) (high-fat diet plus 20 mg/kg protein hydrolysates per body weight of rat), and HFD+HP(300) (high-fat diet plus 300 mg/kg protein hydrolysates per body weight of rat) (34). To create a high-fat diet, 10% animal fat and 5% fructose were added to 85% of the regular diet (35). Daily, freshly produced protein hydrolysates were diluted in 4 ml of distilled water at 20 and 300 mg/kg body weight concentrations of the rats. Using an oro-gastric feeding needle, the solution was given to the rats through their gastrointestinal tract. The control group was given a conventional diet, and 4 cc of distilled water was given orally using an oro-gastric feeding needle, which is vital to highlight. 

***Fasting glucose concentration, insulin level, and tissue preparation***

After 14 hr of food restriction, during which they had unrestricted access to water, the animals’ fasting glucose concentration and insulin levels were assessed on day 70 after NAFLD induction (36). To guarantee minimum stress during the procedure of blood collection and biopsy, the rats were humanely put to sleep using an overdose of sodium pentobarbital anesthesia (90 mg/kg) (37). Blood samples were taken directly from the heart after weighing. Using a commercial kit from Darman Faraz Kave (Esfahan, Iran), a part of the blood was drawn to assess glucose levels, and the remaining blood was transferred to test tubes containing an anticoagulant. The plasma was kept at -80 °C for 10 min after separation using centrifugation at 3500 g to assess insulin and other metabolic markers. Plasma insulin levels were determined using an ELISA method and a kit from the Zelbio Company (Berlin, Germany).

 The testicular tissues were carefully removed from the specimens, and half of the tissue was preserved in Bouin’s fixative before being processed into paraffin blocks by dehydration. Then, using an automated microtome (LKB, UK), slices with a diameter of 5–6 mm were created. Five sections per rat—a total of 15 sections—were then evaluated. Hematoxylin and eosin (H&E) staining was applied to these sections to investigate and interpret the alterations that were found. The percentage of seminiferous tubules with more than 3–4 germinal layers was used to calculate the positive tubular differentiation index (TDI), and the proportion of tubules with normal spermiogenesis was used to calculate the positive SPI index (38).

***OS evaluation in testicular tissue***

0.2 g of previously frozen testicles were homogenized, and the resultant supernatant was collected to measure the tissue’s total anti-oxidant capacity (TAC) level. The technique developed by Lowry was used to determine the total protein level (39). After that, the TAC findings were converted to nmol/mg protein. According to the procedure developed by Khavarimehr *et al*. (2017), the ferric reduction anti-oxidant power (FRAP) assay was carried out, and the absorbance was determined at 593 nm (40). A lipid peroxidation assay kit (Arsam Fara Biot, Urmia, Iran) developed based on the TBARS test was used to measure the amount of malondialdehyde (MDA) in the tissue. The level of malondialdehyde (MDA) in the tissue was evaluated using a lipid peroxidation assay kit (Arsam Fara Biot, Urmia, Iran) based on the TBARS test. At 532 nm, the samples’ absorbance was gauged. A reduced glutathione assay kit (Arsam Fara Biot, Urmia, Iran) based on the DNTB technique and at 412 nm was used to determine the levels of reduced glutathione (GSH). A glutathione test kit (Navand Salamat, Urmia, Iran) was used to detect oxidized glutathione (GSSG), and the absorbance of the samples was determined at a wavelength of 412 nm.

*Immunohistochemical (IHC) staining*

To look at the expression of HSP70, HSP90, and 8-oxodG in testicular tissue, immunohistochemical staining was done. After preparation, sections were deparaffinized, hydrated with ethanol, and PBS-washed. Hydrogen peroxide was used to inactivate endogenous peroxidases before anti-HSP70-2a (1:300, Elabscience, USA, Cat N: E-AB-18739), anti-HSP90 (1:500, Elabscience, USA, Cat N: E-AB-10353), and anti-8-oxodG (1:100, Genox Corporation, USA, Cat N: MOG-020P) primary antibodies were applied to the sections. Sections were treated with a secondary antibody (1:500, Elabscience, USA, Cat N: E-AB1003) coupled to goat anti-rabbit IgG peroxidase/HRP following an overnight incubation at 4 °C. Using diaminobenzidine chromogenic substrate (DAB), the targeted proteins may be seen as a brown tint. Using hematoxylin staining, the nucleus was contrasted. Per particular seminiferous tubule, the number of proteins indicated by brown reactions was measured (19).

***RNA isolation, cDNA synthesis, and qRT-PCR***

According to Mahmoudian *et al*. (2013), RNA was extracted using the TRIZOL technique and examined for quality and concentration (41). A reaction mixture including certain components, such as RNA, was made, and primers for TP1, TP2, HSP90, HSP70-2a, and GAPDH were constructed using the MAFFT tool (Table 1). Using NCBT BLAST software, the primer specificity was verified, and duplicate PCRs were run for each sample group. An RT-PCR machine was used to carry out the reactions, which comprised template cDNA, Cyber Green Master Mix, and reverse and forward primers specific to the desired gene. On a 2% agarose gel, PCR products were seen. By standardizing the mean threshold cycle (Ct) PCR values for the target mRNAs and GAPDH, which were computed using the equation (2^^-(Ct target - Ct GAPDH)^), the relative expression level of the target mRNA was ascertained. 

***Statistical analyse*** *s*

The Kolmogorov-Smirnov test was used to determine whether data variances were normal, and Levene’s test was used to determine whether variances were homogeneous. One-way ANOVA was used in the statistical analysis using SPSS software (version 21.00), followed by the relevant *post hoc* tests and Bartlett’s test. Statistical significance was defined as a *P*-value of 0.05. Every piece of data is displayed as mean ± SD.

## Results

***Protein hydrolysates improved the relative weight of the testis and diminished the fasting glucose concentration and insulin levels***

**Figure 2:**
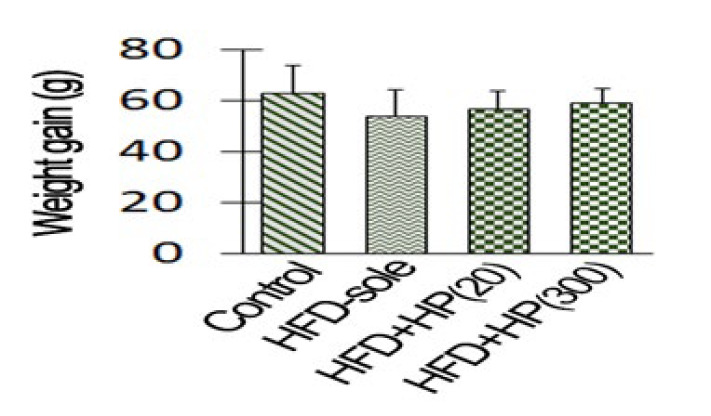
Effects of protein hydrolysates derived from whiteleg shrimp waste on the rats’ weight gain in different groups. IIn the figures, HFDsole

**Figure 3:**
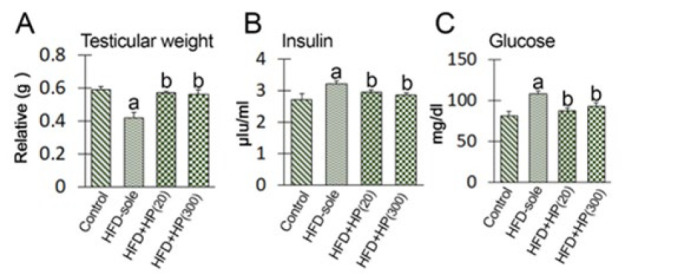
Impact of protein hydrolysates derived from Whiteleg shrimp waste on relative testis weight, serum insulin, and fasting blood glucose levels in different groups of the experiment

The results of the weight gain and relative weight of the testis are presented in Figures 2 and 3. The results indicate a significant reduction in the testicular weight relative to the total body weight in the HFD-sole group compared to the control rats (*P*=0.03). Additionally, the findings of this study demonstrated that the administration of HP, both at low (*P*=0.02) and high (*P*=0.03) concentrations, led to a significant increase in the relative weight of the testis compared to the HFD-sole group. Notably, even at higher concentrations, the increase in testis weight remained significant compared to the control treatment. A consistent pattern of changes was observed in both blood glucose concentration and insulin levels, with the rats in the HFD-sole group exhibiting significant differences compared to the control group in both indicators (*P*=0.002). However, the administration of HP resulted in a significant reduction in glucose and insulin levels compared to those in the HFD-sole group, as evidenced by the obtained results (*P*=0.01).

***HP ameliorated histological damage and improved tubular differentiation and spermiogenesis indices***

**Figure 4:**
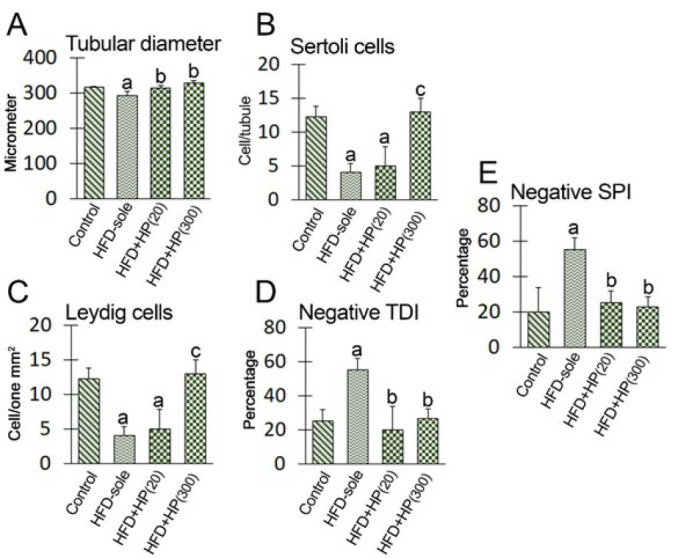
Effects of experimental treatments on seminiferous tubule diameter, mean distributions of Sertoli/seminiferous tubule and Leydig cells/one mm^2^ of tissue, negative TDI, and negative SPI indices in different groups. In the figures, HFD-sole refers to a high-fat diet while HFD+HP (20) represents a high-fat diet + 20 mg/kg protein hydrolysates per body weight of rat, and the HFD+HP (300) group represents a high-fat diet + 300 mg/kg protein hydrolysates per body weight of rat

Figure 4 displays the outcomes regarding the impact of the experimental treatments on histomorphometric changes as well as the mean distributions of somatic (Leydig and Sertoli) cells. As shown in Figure 4, the HFD group exhibited the lowest seminiferous tubule diameter, significantly different from that of the control group (*P*=0.001). Conversely, the findings indicated that HP could improve HFD-induced histological damage. Accordingly, the results revealed no significant difference in the number of Leydig cells across the experimental treatments (*P*>0.05). However, the HFD-sole and HFD+HP(20) groups exhibited a significant reduction in the number of Sertoli cells compared to the control treatment (*P*=0.002), whereas the HFD+HP(300) group exhibited a significantly increased number of Sertoli cells compared to the HFD-sole group (*P*=0.002). No significant difference was observed between the HFD+HP300 and control groups (*P*>0.05).

Accordingly, both HP-treated groups showed increased tubular diameter and lower percentages of tubules with negative TDI and SPI. When comparing the experimental and control groups, no discernible differences in the mean numbers of Leydig cells/mm2 of the interstitial connective tissue were found (*P*>0.05). In contrast, the HFD-sole animals had a considerably (*P*=0.01) lower average distribution of Sertoli cells/seminiferous tubules than the control group. Compared to the HFD-sole group, the HFD+HP(300) group had a noticeably (*P*=0.001) larger distribution of Sertoli cells.

***HP improved anti-oxidant capacity and reduced the lipid peroxidation ratio in the testis***

**Figure 5:**
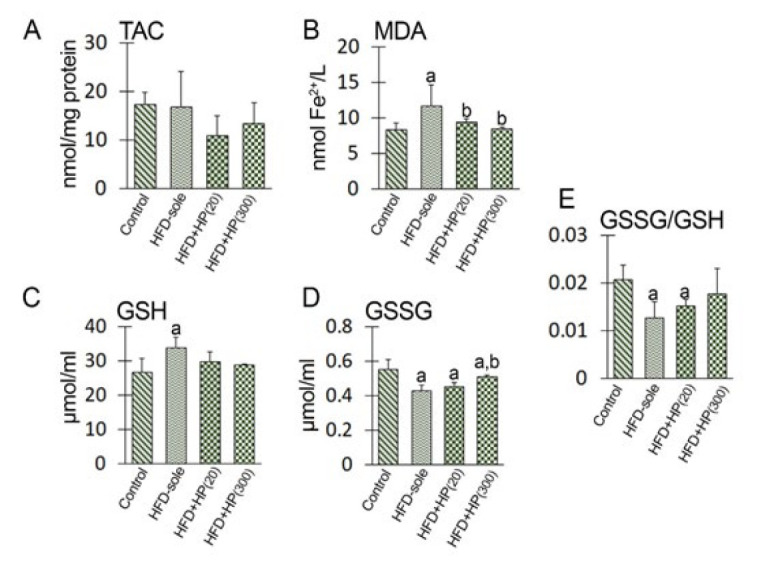
Effects of experimental treatments on the antioxidant capacity of the testis in the rats

The TAC level and MDA content were examined to explore the effect of HFD-induced NAFL on testicular anti-oxidant status and lipid peroxidation ratio. The results are presented in Figures 5A and 4B. No significant change was revealed in the testicular TAC level between the control and the experimental groups (*P*>0.05). The MDA content was significantly (*P*=0.01) increased in the HFD-sole group compared to the control rats. In contrast, the situation was reversed in the groups treated with HP. Both the HFD+HP(20) (*P*=0.027) and the HFD+HP(300) (*P*=0.021) groups showed a significant decrease in MDA content compared to the rats solely fed the high-fat diet (HFD-sole).

***HP improved the GSH/GSSG balance***

To investigate the impact of HFD-induced NAFL on the redox status related to testicular GSH and GSSG, we focused solely on assessing the f GSH and GSSG levels, as well as calculating their relative ratio (GSH/GSSG) in all experimental groups. The results are presented in Figures 5C, 5D, and 5E. Observations revealed a significant (*P*=0.03) increase in the GSH level versus the control rats. No significant (*P*>0.05) changes were shown between the control, HFD+HP(20), and HFD+HP (300) groups. The GSSG levels were significantly decreased in all experimental groups compared to the control rats (HFD-sole: *P*=0.001; HFD+HP (20): *P*=0.02); HFD+HP (300): *P*=0.03). The HFD+HP(300) group presented markedly higher GSSG levels than the HFD-sole rats (*P*=0.04). The analysis of the relative ratio of GSH/GSSG demonstrated that HFD in the HFD-sole group significantly (*P*=0.01) decreased the GSH/GSSG ratio. However, this ratio remained unchanged in the HFD+HP (20) group and significantly increased in the HFD+HP (300) group (*P*=0.001). Notably, no significant difference was observed between the HFD+HP(300) group and the control rats (*P*>0.05).

***HP rebalanced NAFL-induced changes in HSP70-2a expression at both the mRNA and protein levels***

**Figure 6:**
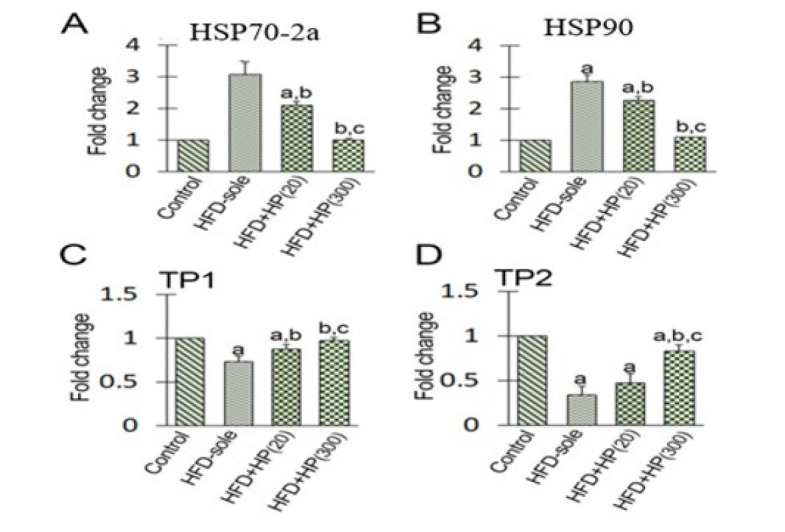
qRT-PCR results for mRNA fold changes are as follows: (A) HSP70-2a, (B) HSP-90, (C) transitional protein-1 (TP1), and (D) transitional protein-2 (TP2)

**Figure 7:**
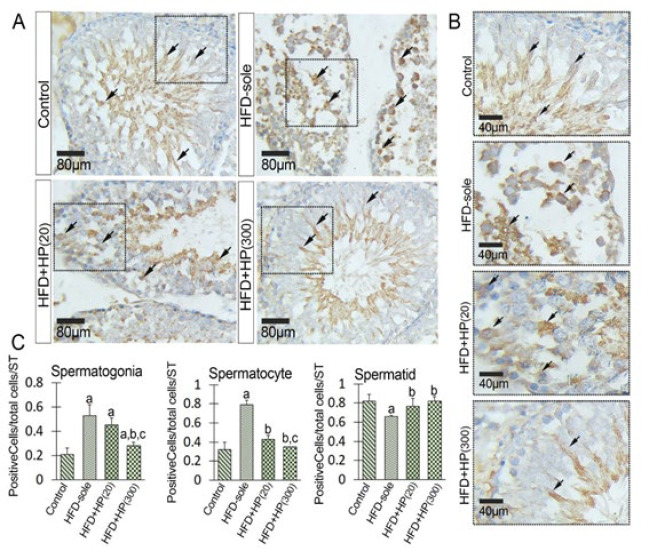
Immunohistochemical staining of HSP70-2a (A) and (B), a cross-section of the seminiferous tubule in the high-fat diet (HFD-sole) group demonstrates an increased distribution of HSP70-2a^+^ cells within the tubule

Given the known homeostatic function of HSP70-2a in various stress conditions and its role in maintaining the chromatin condensation process, we aimed to assess its expression levels (both mRNA and protein) following the administration of HP in the NAFL condition. The mRNA level of HSP70-2a was significantly increased in the HFD-sole group versus the control rats (*P*=0.001). In contrast, HP demonstrated a dose-dependent reduction in the mRNA level of HSP70-2a compared to the HFD-sole group. Specifically, the lowest mRNA levels of HSP70-2a were observed in the HFD+HP(300) (*P*=0.001) and the HFD+HP(20) (*P*=0.01) groups when compared to the HFD-sole rats (Figure 6A). To investigate the changes at the protein level, IHC staining was performed, and the changes in the mean numbers of HSP70-2a^+^ spermatogonia, spermatocytes, and spermatids/seminiferous tubules were assessed. In the HFD-sole group, the mean numbers of HSP70-2a^+^ spermatogonia and spermatocytes were significantly increased (*P*=0.001) compared to those in the control rats. In contrast, the average number of HSP70-2a^+^ spermatids was significantly decreased (*P*=0.01) in the HFD-sole group (Figure 7A, 7B, 7C). HP significantly ameliorated the HFD-induced situation. Specifically, the HFD+HP(20) (*P*=0.002) and HFD+HP(300) (*P*=0.001) groups represented a remarkable reduction in the mean distributions of HSP70-2a^+^ spermatogonia and spermatocytes compared to the HFD-sole rats. Regarding spermatids, both HP-treated groups exhibited an up-regulation of HSP70-2a expression at the protein level, although the differences were not statistically significant between HP-treated groups (*P*>0.05). Accordingly, more HSP70-2a+ cells per tubule were observed in the HFD+HP(20) and HFD+HP(300) groups than in the HFD-sole group.

***HP rebalanced NAFL-induced changes in HSP90 expression at both the mRNA and protein levels***

**Figure 8:**
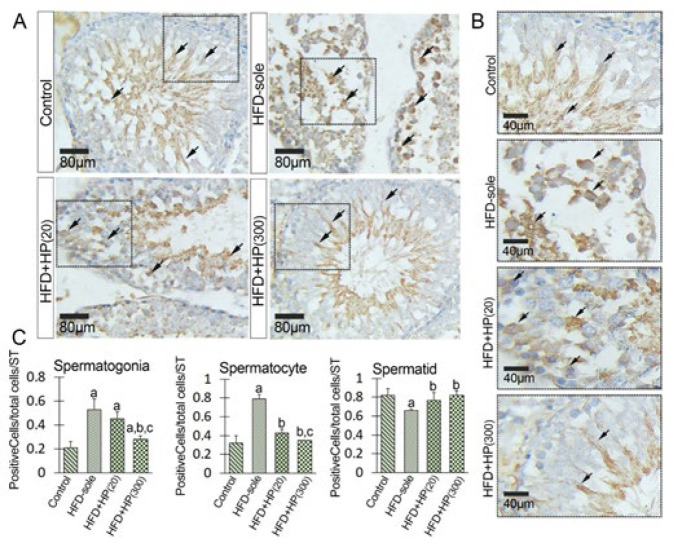
Figure illustrates the immunohistochemical staining of HSP90 (A) and (B), cross-sections of the seminiferous tubules from the HFD-sole and HP-received groups demonstrate an increased distribution of HSP90+ cells within the tubules. Note the increased expression of HSP90 in all series of germinal cells in the HFD-sole group, which is significantly decreased in the hydrolyzed protein-received groups. See the same phenotype in the HFD+HP(300) group compared to the control group cross-sections. (C) presents the quantitative cell count results for HSP90+ spermatogonia, spermatocytes, and spermatids per seminiferous tubule, utilizing consistent criteria across all groups. All data are reported as mean ± SD, (n = 6 rats/group)

Given the known function of HSP90 in various stress conditions and its role in boosting the HSP70-2a-induced role in the chromatin condensation process, we aimed to assess its expression levels (both mRNA and protein) following the administration of HP in NAFL conditions. As shown in Figure 5B, the HFD could significantly (*P*=0.001) increase HSP90 expression at the mRNA level versus the control rats. In contrast, the administration of HP dose-dependently reduced the expression of HSP90 at the mRNA level compared to that in HFD-sole rats. Similarly, the HFD+HP(20) (*P*=0.002) and HFD+HP(300) (*P*=0.001) groups exhibited a remarkable reduction in HSP90 mRNA expression compared to the HFD-sole rats. To examine the changes at the protein level, IHC staining was employed to assess alterations in the mean numbers of HSP90+ spermatogonia, spermatocytes, and spermatids per seminiferous tubule. In the HFD-sole group, the average numbers of HSP90^+^ spermatogonia and spermatocytes were significantly increased (*P*=0.001) compared to those in the control rats, while the mean number of HSP90^+^ spermatids was significantly decreased (*P*=0.02) (Figure 8A, 8B, 8C). However, the administration of high-dose HP (in the HFD+HP(300) group) was able to effectively ameliorate the detrimental effects induced by HFD (*P*=0.001) in comparison to the HFD-sole rats. In terms of spermatids, both HP-treated groups exhibited an increase in HSP90 expression at the protein level, although the differences between the HP-treated groups were not statistically significant (*P*>0.05).

***Protein hydrolysates amplified TP1 and TP2 expression in HFD-fed rats***

The HFD-sole group exhibited a significant reduction in the mRNA levels of TP-1 and TP-2 compared to the control group (*P*=0.001). In contrast, HP administration significantly increased the mean TP1 and TP2 mRNA levels compared to those in HFD-sole rats. Accordingly, the high-dose HP-treated group (HFD+HP(300) group) exhibited no significant differences in TP1 and TP2 mRNA levels versus the control rats (Figure 6C, 6D).

***Protein hydrolysates reduced the HFD immunoreactivity of 8-oxide***

**Figure 9:**
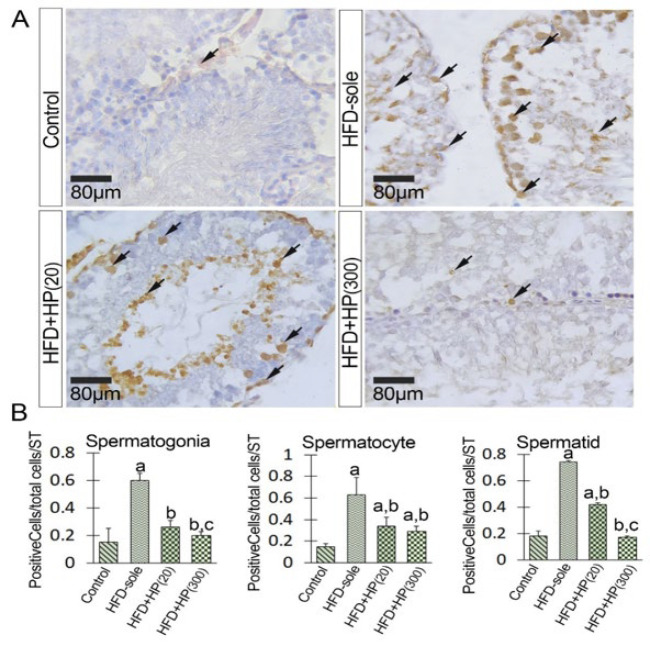
Immunohistochemical staining of 8-oxodG for oxidative DNA damage (ODD); (A) 8-oxodG immunoreactivity in the testes from control, high-fat diet-sole-received and protein hydrolysates groups showing strong staining in the HFD-sole group, which is significantly decreased in the protein hydrolysates-received groups. (B) presents the quantitative cell count results for 8-oxodG+ spermatogonia, spermatocytes, and spermatids per seminiferous tubule, utilizing consistent criteria across all experimental groups

To uncover the possible effect of NAFL conditions on the DNA integrity of germ cells and to examine the protective effect of HP on NAFL-induced oxidative DNA damage, the immunoreactivity of 8-oxodG was assessed. The HFD-sole rats exhibited a remarkable (*P*=0.001) increase in the mean 8-oxodG^+^ spermatogonia, spermatocytes, and spermatid cells relative to total cells per seminiferous tubule compared to the control group. In contrast, HP, in a dose-dependent manner, reduced the 8-oxodG^+^-positive cell distribution compared with that in HFD-sole rats (Figure 9A, 9B). Accordingly, both the HFD+HP(20) and HFD+HP(300) groups showed a remarkable reduction in the mean distributions of 8-oxodG^+^ spermatogonia versus the HFD-sole group (*P*=0.001).

The enzyme-to-substrate ratio used was 5%, and the hydrolysis process was carried out for 180 min at a temperature of 50 °C

**Table 1:** The nucleotide sequences used in the qRT-PCR were applied to analyze the expression of HSP70, HSP90, TP1 and TP2 genes in the testes of the rats

Genes	Forward	Reverse
HSP70 (NM_021863.3)	GACCTGGGCACCACTTAC	TTGGTGGGGATGGTGGAGTTG
HSP90 (XM_008764969.2)	TGGACAGCAAACATGGAGAG	TGTAACCCATTGTTGAGTTGTCT
Transitional protein 1 (NM_017056.2)	TGAGGAGAGGCAAGAACC	ATCGCCCCGTTTTCCTAC
Transitional protein 2 (NM_017057.2)	CGGACTCACAGAGCTAAGAG	TCCCCTAGTGATGGCTATCT
GAPDH (XM_017592435.1)	CTGCACCACCAACTGCTT	GCCATCCACAGTCTTCTG

refers high-fat diet while in the HFD+HP(20) and HFD+HP(300) groups, the rats received HFD supplemented with 20 and 300 mg protein hydrolysates /kg body weight. HFD: High-fat diet; HP: Protein hydrolysate

The data are presented as the mean ± SD (n = 6 per treatment). In the figures, HFD-sole refers to a high-fat diet while HFD+HP(20) represents a high-fat diet + 20 mg/kg protein hydrolysates per body weight of rat, and the HFD+HP(300) group represents a high-fat diet + 300 mg/kg protein hydrolysates per body weight of rat. Different letters in each column indicate significant differences between the diets: the letter "a" denotes a significant difference from the control diet and the letter "b" indicates a significant difference from the HFD-sole diet, all at a 95% confidence level

HFD: High-fat diet; HP: Protein hydrolysate

Significant differences among the diets are indicated by different letters in each column: the letter "a" denotes a significant difference from the control diet, the letter "b" indicates a significant difference from the HFD-sole diet, and the letter "c" denotes a significant difference from HFD+HP (20), all at a 95% confidence level. All data are presented as the mean ± SD (n = 6 per treatment)

TDI: Tubular differentiation index; HFD: High-fat diet; HP: Protein hydrolysate

In the figures, the HFD-SOL refers to the high-fat diet while the HFD+HP (20) group represents the high-fat diet + 20 mg/kg protein hydrolysates per body weight of rat, and the HFD+HP (300) group represents the high-fat diet + 300 mg/kg protein hydrolysates per body weight of rat. Different letters in each column indicate significant differences among the diets: "a" denotes a significant difference from the control group, and letter "b" indicates a significant difference from HFD-sole, all at a 95% confidence level. All data are presented as the mean ± SD (n = 6 per treatment)

TAC: Total antioxidant capacity; MDA: Malondialdehyde; GSH: Glutathione; GSSG: Glutathione disulfide

In the figures, HFD-sole refers to a high-fat diet while the HFD+HP (20) group represents a high-fat diet + 20 mg/kg protein hydrolysates per body weight of rat, and the HFD+HP (300) group represents a high-fat diet + 300 mg/kg protein hydrolysates per body weight of rat. Significant differences among the diets are indicated by different letters in each column: the letter "a" denotes a significant difference from the control diet, the letter "b" indicates a significant difference from the HFD-sole group, and the letter "c" represents a significant difference from the HFD+HP (20) diet, all at a 95% confidence level. All data are presented as the mean ± SD (n = 6 per treatment)

HFD: High-fat diet; HP: Hydrolyzed protein

Notably, condensing spermatids are observed with no positive reaction for HSP70-2a in the HFD-sole group when examining the higher magnification images. However, this situation is significantly ameliorated in the hydrolyzed protein-received groups. (C) presents the quantitative cell count results for HSP70-2a^+^ spermatogonia, spermatocytes, and spermatids per seminiferous tubule, with consistent criteria applied across all groups. Significant differences among the diets are indicated by different letters in each column: the letter "a" denotes a significant difference from the control diet, the letter "b" indicates a significant difference from the HFD-sole group, and the letter "c" represents a significant difference from the HFD+HP(20) diet, all at a 95% confidence level. All data are presented as the mean ± SD (n = 6 per treatment). HFD+HP(20): a high-fat diet + 20 mg/kg protein hydrolysates per body weight of rat, HFD+HP(300): a high-fat diet + 300 mg/kg protein hydrolysates per body weight of rat.

HFD: High-fat diet; HP: Hydrolyzed protein

Significant differences among the diets are indicated by different letters in each column: the letter "a" denotes a significant difference from the control diet, the letter "b" indicates a significant difference from the HFD-sole group, and the letter "c" represents a significant difference from the HFD+HP(20) diet, all at a 95% confidence level. All data are presented as the mean ± SD (n = 6 per treatment). HFD-sole: high-fat-sole received, HFD+HP(20): a high-fat diet + 20 mg/kg protein hydrolysates per body weight of rat, HFD+HP(300): a high-fat diet + 300 mg/kg protein hydrolysates per body weight of rat. HFD: High-fat diet; HP: Hydrolyzed protein

Significant differences among the diets are indicated by different letters in each column: the letter "a" denotes a significant difference from the control diet, the letter "b" indicates a significant difference from the HFD-sole group, and the letter "c" represents a significant difference from the HFD+HP(20) diet, all at a 95% confidence level. All data are presented as the mean ± SD (n = 6 per treatment). Note: HFD-sole: high-fat-sole received, HFD+HP(20): a high-fat diet + 20 mg/kg protein hydrolysates per body weight of rat, HFD+HP(300): a high-fat diet + 300 mg/kg protein hydrolysates per body weight of rat

HFD: High-fat diet; HP: Hydrolyzed protein

## Discussion

As a preliminary finding, we observed a significant increase in serum glucose and insulin in the NAFLD condition induced by HFD. Our histopathological analysis indicates that the NAFLD condition (HFD-sole group) caused testicular atrophy compared to the control group, significantly reducing testicular weight. Moreover, the HFD-sole group displayed a reduction in seminiferous tubule diameter, as well as a decrease in Leydig and Sertoli cell distribution and reduced percentages of seminiferous tubules with progressive germ cell differentiation and spermiogenesis (marked with TDI and SPI indices). Furthermore, our findings demonstrate that HFD consumption significantly perturbs the redox system in the testis. This disruption is accompanied by a notable up-regulation of HSP70-2a and HSP90 expression and a decrease in TP1 and TP2 mRNA levels compared to the control group. Additionally, extensive oxidative DNA damage (ODD) was observed in both germ and somatic cells of HFD-exposed rats. In contrast, the groups that received HP exhibited improvements in TDI and SPI indices and redox status and represented a remarkable reduction in the NAFLD-induced ODD. Moreover, the HP-treated groups showed down-regulated expression of HSP70-2a and HSP90 and amplified TP1 and TP2 expression compared to the HFD-sole group. The current findings align with previous research that has consistently shown the beneficial effects of HP or other anti-oxidants on testicular morphology and dysfunctions associated with metabolic disorders (42).

Indeed, NAFLD contributes to fat accumulation in the liver and testicular tissue, resulting in increased levels of ROS and an imbalance in the redox system (43, 44). Both of these alterations can lead to an imbalanced oxidant/anti-oxidant ratio in the testicles, resulting in oxidative or reductive stress. Although OS is a well-known contributor to testicular damage, reductive stress/imbalance (RS), characterized by an excess accumulation of reducing equivalents, can also lead to significant ODD and lipid peroxidation in the testes (19, 45). The key difference between OS and RS is that, in the case of RS, no significant changes were detected in the TAC. However, there is a pathological increase in GSH levels, which disrupts the balance of the relative GSH to GSSG ratio compared to the physiological state (46). Additionally, an increased content of MDA is observed in RS, indicating massive lipid peroxidation (47, 48). Therefore, given the importance of the oxidant/anti-oxidant balance in the context of NAFLD-related impairment (34), we analyzed testicular TAC, MDA, GSH, and GSSG levels, as well as MDA content, in the treatments. Observations showed no significant changes in the total TAC but a remarkable increase in GSH and MDA levels in the HFD-sole group compared to the control rats. However, the administration of HP led to a significant improvement in these parameters. Specifically, the HFD+HP (300) group exhibited significantly lower MDA and GSH levels and an ameliorated GSH/GSSG ratio relative to the HFD-sole group. Minding the role of the GSH/GSSG conversion system under stress conditions, where the irreversible conversion of GSH to GSSG or vice versa occurs (47) and the triggering effect of GSH overproduction in RS (48), it can be suggested that NAFLD significantly affects the testicular redox system, resulting in RS and its consequents such as lipid peroxidation and ODD in the testes. In line with this issue, the HFD-sole rats exhibited massive ODD (marked with 8-oxodG immunoreactivity) and increased MDA content. Conversely, the decreased levels of GSH, along with an improved GSH/GSSG ratio and reduced MDA content, suggest that the administration of HP, particularly at higher doses, can promote the testicular redox system and inhibit HFD-induced RS. This, in turn, can lead to a reduction in ODD and lipid peroxidation. (49) Our results agree with previous findings that HP and bioactive peptides remove free radiation in tissue environments and protect DNA against ROS (29, 50-52).

It is important to highlight that OS/RS has been shown to significantly impact the expression of testis-specific HSPs as stress responders (21, 22, 45). Among the HSP family, HSP70-2a and HSP 90 play a crucial role in spermatogenesis development by safeguarding the DNA content of haploid cells, the DNA-packaging process, condensation of chromatin structure (20, 23, 53) and even boosting the testicular redox system by maintaining anti-oxidant enzyme expression and/or activity (28). Therefore, considering the crucial roles of HSPs as stress responders in OS and RS conditions (45), we investigated the effects of NAFLD before and after HP administration on the testicular hemostatic status under NAFLD-induced RS conditions. For this purpose, we specifically focused on the heat shock proteins HSP70-2a and HSP90, which are specifically involved in many protective processes in germ and somatic cells in testicular tissue under RS conditions. According to our research, NAFLD (HFD-induced) dramatically increases the mRNA and protein production of HSP70-2a and HSP90. This implies that NAFL-induced RS may cause an increase in HSP expression, possibly as a defense mechanism to prevent RS-induced redox imbalance and preserve hemostasis. By boosting glutathione peroxidase (GPx) and glutathione reductase (GR) activities, HSP70 and HS90, in particular, are implicated in controlling the redox balance (28). About this problem, it has been demonstrated that HSP70-2a prevents ROS and reactive nitrogen species (RNS) from forming while also encouraging the development of anti-oxidant enzymes, including superoxide dismutase (SOD) and catalase (54, 55). HSP90, on the other hand, has been reported to regulate the activity of several key redox-regulated proteins, including nitric oxide synthase (NOS), Akt, and p53, thereby modulating intracellular redox signaling pathways (55, 56). On the other hand, we found that administration of HP could significantly prevent this situation. Accordingly, HP (20), at a lower impact, and HP (300), more effectively, could reduce the HSP (HSP70-2a and HSP90) expression levels compared to the HFD-sole rats. These findings indicate that HP may regulate the redox system by modulating the balance between GSH and GSSG, which sustains the preexisting levels of HSPs without increasing their expression ratio. In line with this issue, previous studies have demonstrated that HP, due to the presence of a high amount of bioactive peptides, possesses the ability to scavenge oxygen radicals, chelate pro-oxidant metal ions, and inhibit lipid peroxidation(57, 58). Considering these properties, it is plausible to suggest that HP, a potent anti-oxidant, interacts with free radicals generated by NAFL, thereby maintaining the enzymatic anti-oxidant capacity of the testes. In essence, HP may be utilized in scavenging ROS, thereby reducing the need for other endogenous anti-oxidant agents to interact significantly, as seen in the HFD-sole group. This hypothesis is supported by our observations of a balanced GSH/GSSG ratio and reduced MDA content in rats that received HP supplementation [HFD+HP(20) and HFD+HP(300)]. Indeed, the overexpression of HSPs (HSP70-2a and HSP90) occurs under OS/RS conditions. However, in the presence of controlled OS/RS conditions (most likely maintained by bioactive peptides in HP), there might be no need for additional HSP expression. During spermatid elongation, protamines replace histones, leading to chromatin condensation and packaging of DNA in sperm. This process requires the coordinated action of various proteins, including TP1 and TP2, which facilitate the movement of histones. Indeed, TP1 and TP2 act as transitional proteins that assist in replacing histones with protamines, which results in proper DNA packaging during spermatogenesis (59, 60). Therefore, specific epigenetic changes are essential to protect the DNA content against oxidative ODD and initiate the gene-silencing process through active methylation (61, 62). HSP70-2a plays a critical function in the histone-protamine exchange process during the postmeiotic stage in testicular tissue, as evidenced by its strong relationships with transition proteins (TP1 and TP2) and other epigenetic processes (19). Similarly, while not identical in function, HSP90 also plays a vital role in chromatin structure. Specifically, HSP90 is involved in the tightening and condensing of chromatin structure, which impacts chromatin organization and heritable gene regulation (19, 23). Given these details, it is imperative to note that the presence of active HSPs and TPs in elongated spermatids is critical for aiding DNA packing and protecting the genome’s integrity against ROS-caused nucleotide damage (ODD). Detailed research was done to determine how an HFD and HP affected the expression of HSP70-2a, HSP90, and TPs (TP1 and TP2) in elongated spermatozoa. The results showed that whereas spermatogonia and spermatocytes had higher levels of HSP70-2a and HSP90 expression, elongated spermatozoa in the HFD group had significantly lower levels of these HSPs’ expression. Furthermore, the HFD-sole group showed a striking decrease in the mRNA levels of TPs (TP1 and TP2). In contrast, HP administration increased the expression of HSPs in elongated spermatozoa and amplified TP expression. Scientific data shows that a decrease in TPs, either alone or in combination with decreased HSP expression levels, can significantly impair the chromatin condensation process (63, 64). It makes sense to hypothesize that HP administration, which can increase the expression levels of these proteins, may aid in maintaining correct chromatin condensation. To comprehend the topic, it is important to remember that chromatin condensation problems can dramatically increase the amount of ROS that DNA nucleotides are exposed to, which can lead to ODD (65, 66). In contrast to the preserved DNA content in the HP-received groups, the higher levels of oxidative DNA damage (ODD) seen in the HFD-sole group show that HP can protect the integrity of sperm DNA against ODD by up-regulating HSPs and TPs in elongated spermatozoa.

## Conclusion

Our study has demonstrated that NAFL induced by an HFD can significantly disrupt the balance of the redox system by impairing the GSH/GSSG balance, ultimately leading to OS in the testicles. In response, the expression of HSPs, including HSP70-2a and HSP90, increases in spermatogonia and spermatocytes to maintain the hemostatic status of these cells. However, the expression of TPs, including TP1 and TP2, decreases in elongated spermatozoa, disrupting the chromatin condensation process and resulting in significant ODD in both germ and somatic cells. Fortunately, the administration of HP, which contains high amounts of bioactive peptides, can boost the redox system and preserve the testicular anti-oxidant status, inhibiting the overgeneration of ROS in NAFL conditions. Moreover, HP can amplify the expression of HSPs and TPs in NAFL conditions, thereby maintaining the chromatin condensation process and safeguarding sperm DNA content against NAFL-induced ODD.

## Declaration

We have not used any AI tools or technologies to prepare this manuscript.
